# From Polio to COVID-19: Examining the Impact of Pandemics on Childhood Vaccination Programs

**DOI:** 10.7759/cureus.39460

**Published:** 2023-05-25

**Authors:** Divyansh M Budhia, Arpita Jaiswal, Roshan Prasad, Seema Yelne, Mayur B Wanjari

**Affiliations:** 1 Medicine, Jawaharlal Nehru Medical College, Datta Meghe Institute of Higher Education and Research, Wardha, IND; 2 Obstetrics and Gynaecology, Jawaharlal Nehru Medical College, Datta Meghe Institute of Higher Education and Research, Wardha, IND; 3 Medicine and Surgery, Jawaharlal Nehru Medical College, Datta Meghe Institute of Higher Education and Research, Wardha, IND; 4 Nursing, Shalinitai Meghe College of Nursing, Datta Meghe Institute of Higher Education and Research, Wardha, IND; 5 Research and Development, Jawaharlal Nehru Medical College, Datta Meghe Institute of Higher Education and Research, Wardha, IND

**Keywords:** global collaboration, vaccine hesitancy, covid-19, polio, childhood vaccination, pandemics

## Abstract

This review article aims to examine the impact of pandemics on childhood vaccination programs, specifically looking at the examples of polio and coronavirus disease 2019 (COVID-19). The article provides a comprehensive overview of the history of polio vaccination programs, including the challenges of eradicating the disease and the barriers to vaccine uptake. The article also looks at the global efforts to eradicate polio, such as the Global Polio Eradication Initiative, and the progress made in reducing the number of polio cases worldwide. The article reviews the impact of the COVID-19 pandemic on childhood vaccination programs and how the pandemic has disrupted routine vaccination services. Lockdowns and travel restrictions have contributed to this, which has reduced access to medical facilities and vaccine uptake. The article also explores how the prioritization of COVID-19 vaccines has led to a diversion of resources away from routine childhood immunization programs. The article highlights the need to address these challenges to prevent a resurgence of vaccine-preventable diseases. Furthermore, the article discusses the lessons learned from these pandemics, such as the importance of global collaboration, vaccine equity, addressing vaccine hesitancy, pandemic preparedness, and embracing technology. The article emphasizes the need to prioritize vaccine equity and ensure that vulnerable populations have access to vaccines. Additionally, the article stresses the importance of addressing vaccine hesitancy and providing effective communication and education about vaccines. The article also advocates for pandemic preparedness, emphasizing the need to invest in research and development of vaccines for emerging infectious diseases. Finally, the article suggests embracing technology as a means to improve vaccine accessibility and distribution.

## Introduction and background

Childhood vaccination programs are a cornerstone of public health efforts around the world. These programs have been instrumental in preventing various infectious diseases, from polio to measles, and have saved countless lives. However, the emergence of pandemics has posed significant challenges to vaccination programs and public health efforts [1-3]. The historical context of vaccination programs highlights their importance in preventing disease and promoting public health. In the mid-20th century, polio was a major public health threat, causing paralysis and death in children worldwide. The development of the polio vaccine and widespread vaccination programs led to the eradication of polio in many countries, demonstrating the power of vaccination to combat disease [3-5].

More recently, the COVID-19 pandemic has significantly impacted vaccination programs for children and adolescents. The pandemic has disrupted routine vaccination services, decreasing vaccination coverage and potentially putting children and adolescents at risk of vaccine-preventable diseases. This has highlighted the importance of maintaining vaccination programs during pandemics and other public health emergencies [1-3]. 

The primary objective of the review article is to analyze and assess the impact of pandemics, with specific emphasis on the shift from the polio era to the ongoing COVID-19 pandemic, on childhood vaccination programs. The article endeavors to conduct a comprehensive exploration of how pandemics have affected the accessibility, coverage, and overall efficacy of immunization initiatives for children on a global scale.

Ultimately, the review article strives to contribute to the existing knowledge base on this topic, fostering an enhanced comprehension of the challenges and opportunities presented by pandemics within the realm of childhood vaccination programs. It aims to facilitate evidence-based decision-making, promote the implementation of effective interventions, and safeguard the progress made worldwide in reducing vaccine-preventable diseases.

## Review

Methodology

**Figure 1:**
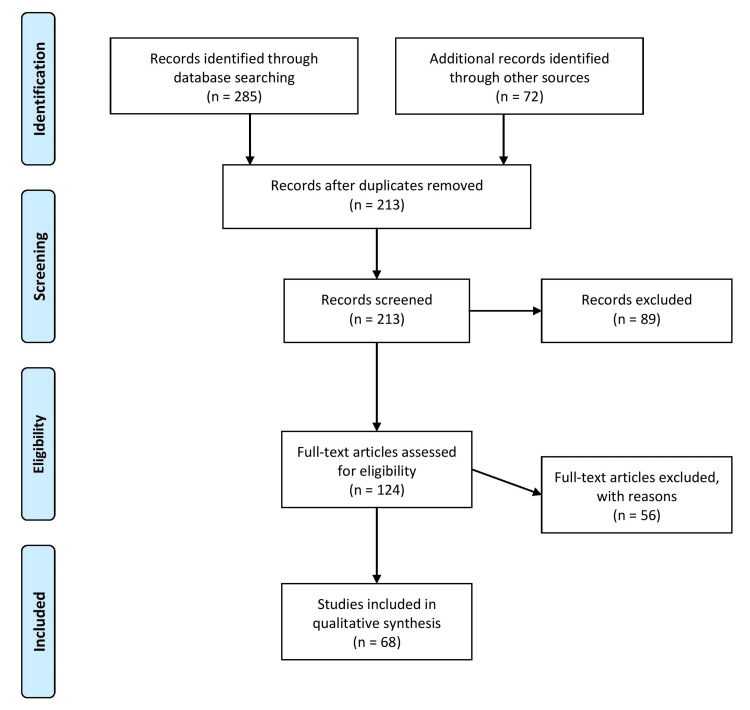
The selection process of articles used in this study. Adopted from the Preferred Reporting Items for Systematic Reviews and Meta-Analyses (PRISMA).

The methodology for this review article includes a comprehensive literature search of academic databases, such as PubMed, Web of Science, and Scopus, to identify relevant articles, reviews, and reports related to the impact of pandemics on childhood vaccination programs. The search encompassed articles published from 2010 to the present. It utilized specific keywords such as "global collaboration", "vaccine hesitancy", "covid-19", "polio", "childhood vaccination" and "pandemics". Relevant articles, reviews, and reports addressing the research question are selected based on the inclusion and exclusion criteria. The inclusion criteria required that the articles be published in the English language, report on the impact of polio on vaccination programmes around the world, report on the impact of the coronavirus disease 2019 (COVID-19) pandemic on vaccination programmes, report on both observational and interventional studies, and published from 2010 to present, and that the articles were not duplicates. Exclusion criteria included duplicate articles, articles in languages other than English, articles published in non-peer-reviewed journals, and articles published before 2000. The next step involves extracting relevant data and information from the selected articles, reviews, and reports, such as the history of childhood vaccination programs, the challenges faced by vaccination programs during pandemics, and the lessons learned from these pandemics. This process was aimed at analyzing and synthesizing the extracted data to identify common themes, trends, and patterns related to the impact of pandemics on childhood vaccination programs, which were incorporated into the review article. Figure 1 describes the selection process of articles used in our study.

The impact of polio on vaccination programs

Polio has had a significant impact on vaccination programs around the world. Following are some ways in which polio has affected vaccination programs:

Focus on Polio Eradication

The effort to eradicate polio has been a major focus of vaccination programs for several decades. This effort has been significant, requiring many resources and attention from governments, international organizations, and public health professionals. The Global Polio Eradication Initiative (GPEI) was launched in 1988 to eradicate polio through vaccines [6]. And, this initiative made significant progress in reducing the number of polio cases worldwide, with a 99.9% reduction in the number of cases reported from 1988 to 2020 [7].

Global focus on eradicating polio had unintended consequences for other vaccine-preventable diseases by diverting resources. The resources and attention required for polio eradication have sometimes diverted resources away from other vaccine-preventable diseases, such as measles, pertussis, and diphtheria [8]. As a result, these diseases have caused outbreaks in some regions of the world, particularly in countries with weak health systems and low vaccination coverage [9].

In addition, the focus on polio eradication has led to vaccine hesitancy and resistance in some communities, further undermining efforts to control other vaccine-preventable diseases. Some communities have expressed concerns about the safety and efficacy of vaccines and have refused to vaccinate their children, resulting in preventable disease outbreaks [4,10,11].

Increased Vaccine Hesitancy

Vaccine hesitancy refers to reluctance or refusal to vaccinate despite availability due to various reasons or concerns and was one of the most significant challenges these programs encountered in some communities [12]. Despite the significant successes of polio vaccination programs, they have faced various challenges. This issue has arisen for various reasons, including concerns about the safety and efficacy of vaccines or cultural beliefs and practices that discourage vaccination [13,14].

Some communities have been concerned that vaccines may have adverse side effects or be ineffective. These concerns increase due to misinformation about vaccines, disseminated through various channels, including social media [15]. Cultural beliefs and practices have also contributed to this. For example, in some regions, vaccines are associated with Western medicine and are viewed with suspicion. Some people believe that vaccines may alter a child's personality or interfere with the natural development of the immune system [16]. These challenges are not limited to polio vaccination programs and have also been observed in other childhood vaccination programs [17].

Challenges in Reaching Remote Populations

Reaching remote populations during polio vaccination campaigns has been a major challenge for various reasons, including limited infrastructure and resources, geographical barriers, and inadequate healthcare services [18]. Vaccination campaigns with innovative strategies were planned to overcome these challenges to reach the target populations. For example, mobile teams travelled to remote areas to vaccinate children. They successfully got remote people, especially in areas with limited healthcare services [19].

Another strategy was to engage with community leaders. In many cases, they played a vital role in promoting vaccination campaigns and encouraging parents to vaccinate their children [20]. The involvement of community leaders resulted in increased vaccine coverage, and they also helped overcome vaccine hesitancy in some communities. This approach was more effective in areas where there is mistrust of vaccines due to cultural or religious beliefs [21].

Coordination With Multiple Stakeholders

Eradicating any disease is a complex and challenging task that requires the coordination of multiple stakeholders, including governments, international organizations, and non-governmental organizations (NGOs). They play different roles, from providing financial resources to implementing vaccination campaigns. Thus, it is essential to have effective communication and collaboration among them to achieve success [22]. Governments are responsible for providing the necessary infrastructure to support vaccination campaigns while playing a critical role in ensuring sufficient funding and resources for vaccination programs [23].

International organizations like World Health Organization (WHO) and UNICEF provide technical expertise, guidance, and financial support to countries facing challenges implementing vaccination campaigns [24]. NGOs often work on the ground, implementing vaccination campaigns and raising awareness about the importance of vaccination [25]. Effective coordination among these stakeholders is essential for a successful vaccination program. Governments must work closely with international organizations and NGOs to ensure vaccination campaigns are effectively planned and implemented. They must also engage with communities to build trust and address any concerns they may have about vaccination [26].

Lessons for Other Vaccination Programs

The efforts to eradicate polio gave important lessons that can be applied to other vaccination programs. One significant lesson is the importance of coordination among stakeholders involved in vaccination programs, including governments, health organizations, and community groups. This coordination is essential in delivering vaccines to remote and underserved communities [27]. Another lesson is the significance of community engagement, as vaccination programs' success often depends on local communities' involvement and support [28].

While the focus on polio eradication has sometimes diverted resources away from other vaccine-preventable diseases, the global effort to eradicate polio also provided important lessons for other vaccination programs, which will help improve the effectiveness and efficiency of future vaccination programs, ultimately preventing the spread of vaccine-preventable diseases and improving global public health [29].

The impact of the COVID-19 pandemic on vaccination programs

The COVID-19 pandemic has had a significant impact on vaccination programs around the world and the following are some ways in which the pandemic has affected vaccination programs:

Disruption of Vaccination Services

The COVID-19 pandemic has significantly impacted the provision of routine vaccination services in many countries around the world. To contain the spread of the virus, measures such as lockdowns, travel restrictions, and social distancing had been implemented disrupting essential health services, including vaccination programs. As a result, many children have missed their routine vaccinations, which could lead to outbreaks of vaccine-preventable diseases. The disruption of vaccination services during pandemics has serious public health implications, as it could lead to outbreaks of vaccine-preventable diseases such as measles, polio, and pertussis [1,30-34].

The disruption of vaccination services during pandemics has also been attributed to redirecting resources to respond to the pandemic. Health workers, supplies, and funding have been redirected to COVID-19 response efforts, which has led to the deprioritization of routine immunization programs. Additionally, many parents have been hesitant to take their children to health facilities for fear of contracting COVID-19, leading to a further decrease in vaccinated children [20,22,34].

It is essential to mitigate the impact of the pandemic on routine immunization programs by implementing strategies such as maintaining essential health services, providing alternative ways of accessing vaccination services, and addressing vaccine hesitancy. Such strategies will ensure that children receive the essential vaccines they need to protect them from vaccine-preventable diseases, even during pandemics [3,15,29].

Decreased Demand for Vaccines

The COVID-19 pandemic has decreased the demand for vaccines, including those unrelated to COVID-19. One reason for this decline in demand is the fear of contracting COVID-19 when visiting healthcare facilities. With the COVID-19 virus spreading rapidly, many have become wary of going to hospitals, clinics, and other healthcare facilities for routine check-ups, screenings, and vaccinations. This fear has led to a decline in the number of people seeking healthcare services, including childhood vaccinations [35,36].

Moreover, misinformation and conspiracy theories about vaccines have contributed to vaccine hesitancy among some populations, leading to a further decrease in vaccine demand [37]. The closure of some healthcare facilities and the suspension of routine vaccination programs have made it difficult for people to receive vaccines. This is especially problematic in low-and middle-income countries, where access to vaccines was already limited before the pandemic [38].

Shortages of Vaccine Supplies

The COVID-19 pandemic caused significant disruptions in global supply chains, including those related to vaccine manufacturing and distribution, which made it difficult for countries to secure adequate supplies of vaccines, thus creating shortages of some vaccines. The high demand for COVID-19 vaccines also put pressure on the supply of other vaccines, further exacerbating the problem of shortages [39,40]. This shortage created serious consequences for childhood vaccination programs. For example, they led to delays in vaccine delivery or reduced available doses, so the children were left unprotected against preventable diseases. The impact of these shortages was severe in low-and middle-income countries, where childhood vaccination programs are already under-resourced and fragile [4,10-12].

WHO and other global health organizations have been addressing the issue of vaccine shortages by equitable vaccine distribution and developing manufacturing capacities in low- and middle-income countries. Some countries have implemented measures to ensure availability to the most needy but vaccine shortage remains a significant challenge as highlighted by COVID-19. The need for increased investment in vaccine research with the importance of resilient vaccine supply chains [25,41].

Repurposing of Healthcare Resources

The COVID-19 pandemic put much strain on healthcare systems worldwide, necessitating a reallocation of resources to handle the pandemic. As a result, employees, equipment, and facilities were moved away from normal healthcare services, like- immunisation programmes. Childhood immunisation programmes were halted, delayed, or discontinued in many regions worldwide due to this budget reallocation [42]. This was a major concern, as low vaccination coverage rates can lead to outbreaks of vaccine-preventable diseases. These outbreaks will increase morbidity and mortality, particularly in vulnerable populations, including young children, the elderly, and immunocompromised individuals [43].

The repurposing of healthcare resources has been necessary to respond to the urgent needs of the COVID-19 pandemic. Healthcare facilities have been converted into COVID-19 treatment centers, and healthcare personnel have been redirected to care for COVID-19 patients. Additionally, personal protective equipment (PPE) and other essential medical supplies for routine healthcare services, including vaccination programs, have been redirected to address the immediate needs of the pandemic [44].

Delayed introduction of new vaccines

The COVID-19 pandemic has considerably influenced vaccination programmes, with the delayed rollout of new vaccinations being one of the biggest obstacles. Clinical trials, regulatory approvals, and other procedures necessary to launch novel vaccinations have been interrupted. Due to the epidemic, significantly less funding and attention to the development of new vaccines since the priority was to deal with the current crisis [45].

Travel restrictions and other social isolation measures also impacted the clinical trials for novel vaccinations, making enrolling people and carrying out studies challenging. As regulatory bodies have moved their emphasis to approvals connected to COVID-19, there has also been a delay in the regulatory licensing of novel vaccinations. New diseases that may have been averted with new vaccines may not have been discovered in time due to the delayed introduction of new vaccines, which could have long-term effects on global health. Additionally, it can impede efforts to meet international immunisation goals and raise the danger of disease outbreaks [46].

Public health responses to vaccination challenges during pandemics

During a pandemic, public health responses to vaccination challenges can vary depending on the nature and severity of the outbreak and some common responses are as follows.

Maintaining Routine Vaccination Services

Routine immunisation programmes must be maintained to protect vulnerable people from diseases that can be prevented by vaccination. Due to travel limitations and the shifting of resources to pandemic response activities, this work may become difficult during a pandemic. Despite these issues, efforts should be taken to maintain public access to routine vaccinations. Public awareness of the value of routine vaccination during a pandemic can also persuade people to prioritise these services and lessen vaccine reluctance. Overall, it is crucial to maintain routine vaccination services during a pandemic to ensure that the gains made in preventing vaccine-preventable diseases are not reversed [47].

Prioritizing High-Risk Groups

This ensures that those most vulnerable to the disease receive protection first. Such prioritization is often necessary due to the limited vaccine supply. During the COVID-19 pandemic, healthcare workers were identified as a priority group due to their increased risk of exposure. After that, older adults and those with underlying health conditions were prioritized due to their increased risk of developing severe illness or complications from COVID-19 [48]. This strategy has also been used during other pandemics, like the H1N1 influenza pandemic in 2009. During this, pregnant women, children, and individuals with underlying health conditions were prioritised for vaccination [49].

High-risk groups must be prioritised while carefully weighing ethical, practical, and logistical factors. Giving some populations priority access to vaccines may raise ethical and practical questions, like making sure the people who should get the vaccine first are identifiable and can get it. To guarantee that the vaccine reaches those who need it the most, logistics around its distribution, storage, and administration must be carefully controlled [50].

Rapidly Scaling Up Vaccine Production

To ensure enough vaccine doses available to fulfill the rising demand for vaccines during a pandemic, public health authorities must quickly scale up vaccine production. Scaling up vaccine production involves several steps, including identifying pharmaceutical companies with the capacity to increase production, investing in new technologies that can speed up the manufacturing process, and increasing the availability of necessary resources [51,52].

Partnerships between public health authorities and pharmaceutical companies are often established to increase vaccine production capacity and involve agreements such as developing new manufacturing facilities, acquiring new equipment, and additional staff. These partnerships can streamline the manufacturing process, ensuring adequate vaccine doses available to meet the population's needs. Investing in new technologies is another approach to rapidly scaling up vaccine production. New technologies, such as RNA vaccines, can significantly reduce the time required to develop and produce vaccines. These technologies also offer the potential for more efficient and cost-effective vaccine production, which can help reduce the pandemic burden on public health systems. It is essential to ensure the availability of necessary resources such as raw materials, equipment, and personnel. Public health authorities may need to work with suppliers and other stakeholders to secure the necessary resources and ensure they are distributed efficiently [51,52].

Developing New Vaccines

Viruses can mutate and change during pandemics, creating new strains that can evade existing vaccines. To address this challenge, there may be a need to develop new vaccines that can target these emerging strains. Public health authorities may work with researchers and pharmaceutical companies to develop new vaccines that can be quickly tested and brought to market [53].

Developing a new vaccine involves a complex process that includes research and development, preclinical testing, and clinical trials. In some cases, vaccines can be developed relatively quickly, such as the mRNA vaccines developed for COVID-19, which were developed in less than a year [54].

However, developing new vaccines can be time-consuming and expensive, and it may take several years before a new vaccine is available. To accelerate the process, public health authorities may provide funding and regulatory support for developing new vaccines and facilitate collaborations between researchers and pharmaceutical companies [45,47,51,55].

A new vaccine must undergo rigorous testing after its development to ensure it is safe and effective. This involves preclinical testing in animals and three phases of clinical trials in humans. If the vaccine is safe and effective, it can be approved by regulatory agencies for use in the general population [20,22].

Increasing Vaccine Uptake

Increasing vaccine uptake is crucial to controlling the spread of a pandemic and achieving herd immunity. This can be achieved by developing public health campaigns, encouraging people to vaccinate, and addressing vaccine hesitancy through education and communication. Addressing vaccine hesitancy can significantly impact vaccine uptake and, ultimately, the effectiveness of vaccination programs. Public health responses to vaccination challenges during pandemics require a coordinated effort from multiple stakeholders, including governments, pharmaceutical companies, healthcare providers, and the general public. Governments can provide funding and support for vaccine development and distribution, while pharmaceutical companies can work to produce and distribute vaccines promptly and efficiently. Healthcare providers can educate patients and administer vaccines, and the general public can help by getting vaccinated and following public health guidelines [56,57].

The way forward: lessons learned and future directions

The COVID-19 pandemic has highlighted the importance of vaccination programs in global health. Here are some lessons learned and future directions for vaccination programs:

Importance of Global Collaboration

The COVID-19 pandemic has underscored the critical importance of global collaboration in addressing public health emergencies. The development and distribution of vaccines to combat the pandemic required a coordinated effort among governments, international organizations, and the private sector across the globe. Many countries have successfully secured vaccine supplies through bilateral agreements, while others have benefited from multilateral initiatives such as the COVAX facility. In addition, the scientific community has come together to share knowledge and collaborate on research, which has accelerated vaccine development and rollout [58].

The lessons learned from the pandemic have important implications for childhood vaccination programs. Economic, social, and political factors in many parts of the world restrict access to vaccines, disproportionately affecting vulnerable populations. Global collaboration was crucial in addressing these disparities and ensuring vaccines were accessible to all, particularly those in low-and middle-income countries. Collaboration can involve sharing expertise and resources, building vaccine production and distribution capacity, and coordinating efforts to overcome logistical and regulatory barriers [59].

Effective global collaboration also requires a commitment to equity and social justice. It was important to recognize and address the historical and structural factors that had contributed to inequities in vaccine access, such as systemic racism, poverty, and geopolitical power imbalances. By working together, governments, international organizations, and the private sector can create a more equitable and just global health system that prioritizes the needs of all people, regardless of their socio-economic status or geographic location [60,61].

Need for Vaccine Equity

The COVID-19 pandemic has revealed inequities in access to healthcare and vaccines, with marginalized communities disproportionately affected by the virus. The disparities in access to healthcare and vaccines have been driven by a complex set of factors, including poverty, social exclusion, discrimination, and inadequate healthcare infrastructure. These factors have contributed to low vaccination rates in certain populations, with the most vulnerable and marginalized communities often being the last to receive vaccines [12,58,59].

Going forward, there is a need for vaccine equity to address these inequities and ensure that vulnerable populations have access to vaccines. This requires a multi-faceted approach, including increased vaccine production and distribution, improved access to healthcare infrastructure, and community outreach efforts to educate and engage populations that may be hesitant to receive vaccines. In addition, policymakers and public health officials need to prioritize the equitable distribution of vaccines nationally and globally to ensure that all individuals have access to life-saving vaccines regardless of their socio-economic status or geographic location [39,45,51].

Without equitable access to vaccines, the pandemic will devastate communities, particularly those already marginalized and vulnerable. By prioritizing vaccine equity, we can help ensure that all individuals have access to the healthcare resources and protections they need to stay healthy and safe. This protects individual health and contributes to the collective global effort to end the pandemic and build more resilient health systems for the future [39,58,62].

Importance of Addressing Vaccine Hesitancy

The success of vaccination campaigns was severely impacted by vaccine reluctance, particularly during pandemics. Vaccine hesitancy describes the delay or rejection of vaccinations despite the availability of vaccination services. The COVID-19 pandemic has brought attention to the need for immunisation programmes to combat vaccine reluctance [57,63]. Vaccine uptake has been significantly hampered by dissemination of false information and conspiracy theories concerning vaccines. Numerous people's doubts regarding the effectiveness and safety of vaccines might foster suspicion and mistrust of vaccination campaigns. Due to the dissemination of falsehoods and misleading information on social media during the COVID-19 pandemic, these worries have worsened [64].

To resolve the concerns raised above and boost vaccine uptake, immunisation programmes should prioritise education and communication. Building trust in vaccination programmes and addressing vaccine hesitancy can be achieved through effective communication tactics adapted to particular demographics. This can involve dispelling widespread myths and worries and offering precise information regarding the efficacy and safety of vaccines. By being upfront and honest with the public, healthcare professionals and public health officials can also significantly reduce vaccine reluctance. Healthcare professionals can address concerns and promote vaccine uptake by fostering trust and offering correct information [64].

Need for Pandemic Preparedness

The COVID-19 pandemic has demonstrated the critical importance of pandemic preparedness. The global health crisis has exposed weaknesses in public health systems, vaccine development, and supply chain management that have hindered the global response to the outbreak. This has resulted in significant human and economic costs [65].

As we move ahead, pandemic preparation must be given top priority in immunisation programmes. To create and implement efficient plans to stop and manage upcoming pandemics, there must be a coordinated worldwide effort. Research and development of vaccines for newly developing infectious illnesses is one of the essential elements of pandemic preparedness. This entails investing in cutting-edge technology and supporting research on new vaccine delivery systems and platforms. Prioritising the creation of reliable systems for vaccination administration, supply chain management, and distribution. This includes providing appropriate vaccine stocks, training and educating healthcare professionals and creating efficient communication plans to encourage vaccination uptake and manage vaccine hesitancy [66].

Embracing Technology

The COVID-19 pandemic has significantly altered many aspects of the healthcare sector, including how vaccination programmes are created, disseminated, and implemented. One of the most important lessons learned from the pandemic is technology's importance in increasing vaccination accessibility and delivery. For instance, telemedicine has made it possible for medical professionals to connect with patients in far-off locations and keep track of their health status, making it simpler to administer vaccines to those who might otherwise have had trouble getting them [67].

In addition, developing mRNA vaccines for COVID-19 has showcased the potential of using advanced technologies to create effective and safe vaccines in record time. With the availability of these technologies, future vaccine development could be faster and more efficient, producing more effective vaccines [68].

An important lesson was the value of global collaboration. Highlighted by the COVID-19 pandemic in addressing the importance of international cooperation in addressing public health challenges, including vaccine development and distribution. Global collaboration could improve vaccine equity, allowing vulnerable populations to access vaccines in low-income countries with limited resources. Addressing vaccine hesitancy was also a crucial lesson. Vaccine hesitancy has been identified as a major challenge to vaccination programs globally, including during the COVID-19 pandemic. Therefore, vaccination programs must develop strategies to address vaccine hesitancy, such as public health education campaigns, community engagement, and clear and accurate communication about vaccine safety and efficacy. Finally, the COVID-19 pandemic has underscored the importance of pandemic preparedness. Developing a pandemic preparedness plan, including the stockpiling of essential supplies and the creation of a robust public health infrastructure, could help mitigate the impact of future pandemics [67,68].

## Conclusions

Childhood vaccination programs are critical to promoting public health, and pandemics can pose significant challenges to these programs. The historical context of vaccination programs, from polio to COVID-19, demonstrates the importance of vaccination in protecting public health. The impact of pandemics on vaccination programs can be devastating, with reduced vaccination coverage potentially leading to outbreaks of preventable diseases. Public health responses to vaccination challenges during pandemics require innovative approaches to vaccine delivery, public health preparedness, and collaboration between governments, healthcare providers, and communities. By learning from past experiences and implementing effective strategies, we can ensure that vaccination programs continue to protect the health of children and adolescents during pandemics and beyond. Moving forward, there is a need for continued vigilance and preparedness in the face of pandemics and other public health challenges. Maintaining vaccination coverage during pandemics is essential, and continued innovation in vaccination delivery can help overcome the challenges posed by pandemics. Public health professionals and policymakers are critical to promoting childhood vaccination programs and protecting public health.
